# Hypoparathyroidism – management, information needs, and impact on daily living from the patients’ perspective: results from a population-based survey

**DOI:** 10.1007/s42000-023-00459-1

**Published:** 2023-06-28

**Authors:** Matthias Büttner, Dieter Krogh, Dagmar Führer, Carmina Teresa Fuß, Holger Sven Willenberg, Markus Luster, Susanne Singer, Heide Siggelkow

**Affiliations:** 1 Institute of Medical Biostatistics, Epidemiology and Informatics (IMBEI), University Medical Center Mainz, Obere Zahlbacher Straße 69, 55131 Mainz, Germany; 2 University Cancer Centre, Mainz, Germany; 3 Netzwerk Hypopara im Bundesverband Schilddrüsenkrebs – Ohne Schilddrüse leben e.V., Berlin, Germany; 4 Department of Endocrinology, Diabetes and Metabolism and Division of Laboratory Research, University Hospital Essen, University of Duisburg-Essen, Essen, Germany; 5 Division of Endocrinology and Diabetes, Department of Medicine I, University Hospital, University of Würzburg, Würzburg, Germany; 6 Division of Endocrinology and Metabolism, Rostock University Medical Center, Rostock, Germany; 7 Department of Nuclear Medicine, University Hospital Marburg, Marburg, Germany; 8 Clinic of Gastroenterology, Gastrointestinal Oncology and Endocrinology, University of Göttingen, Göttingen, Germany; 9 MVZ Endokrinologikum Göttingen, Göttingen, Germany

**Keywords:** Hypoparathyroidism, Management, Information, Impairment, Patient perspective

## Abstract

**Purpose:**

Hypoparathyriodism (hypoPT) is a rare endocrine disorder. It is not known how hypoPT is managed in Germany or whether patients have unmet information needs or impairments in their daily living.

**Methods:**

HypoPT patients at a minimum of 6 months’ post-diagnosis were invited to participate in an online survey through their treating physician or through patient organizations. An extensive questionnaire, which was developed and pilot-tested with hypoPT patients, was administered.

**Results:**

A total of 264 patients with a mean age of 54.5 years (SD: 13.3), 85.2% female and 92% with postsurgical hypoPT, participated in the study. In total, 74% of the patients reported regular monitoring of serum calcium at least every 6 months, with lower control frequencies for phosphate (47%), magnesium (36%), creatinine (54%), and parathyroid hormone (50%), and 24-h urine calcium excretion (36%) on a yearly basis. Information on symptoms of hypo- and hypercalcemia was available in 72 and 45% of the patients. Information needs were related to the disease and its treatment as well as to nutrition, physical activities/sports, and support opportunities. Statistically significant differences for all information needs in association with symptom burden were observed. Hospitalization for hypocalcemia was reported by 32%, nutritional impairments (38%) or impact on work ability (52%) was available among patients with hypoPT.

**Conclusion:**

HypoPT patients experience impairments in daily living and report unmet information needs. Patient and physician education regarding hypoPT is one of the key concepts for improving the management of patients with hypoPT.

## Introduction

Hypoparathyroidism (hypoPT) is a rare endocrine disorder defined by hypocalcemia with inappropriately normal or low parathyroid hormone levels [1]. The majority of hypoPT cases are caused by neck surgery, other clinical causes being autoimmune, genetic, or idiopathic [2]. Different prevalence rates are reported for different countries, ranging from 9.4 per 100,000 to 37 per 100,000 inhabitants [3–6]. Within the last few years, various guidelines and consensus statements regarding the management and treatment of hypoPT have been published [2, 7–12], including calcium supplementation in combination with active vitamin D as conventional treatment. New treatment options such as synthetic PTH analog (PTH 1–34) and human recombinant parathormone (PTH 1–84) are only available for those patients in whom hypocalcemia cannot be adequately treated with conventional treatment. To date, few studies have investigated whether these recommendations are followed in clinical practice [13–17]. Monitoring patients at regular time intervals is important to assess the blood level of relevant parameters but also to ensure that they are not burdened by symptoms, have quality of life (QoL) impairments, or are at risk of complications or secondary diseases [8, 9, 18]. In addition to regular monitoring, guidelines recommend that information should be provided to the patient. It has been shown that patients with hypoPT have unmet information needs, but this has been investigated in only a few studies [9, 14, 15]. In addition to symptom burden and impairments in QoL, hypoPT has been demonstrated to have a negative impact on daily living. Hospitalizations and emergency room visits due to hypo- or hypercalcemia are not uncommon [15, 19–24]. However, a number of additional factors may impact daily life in patients with hypoPT, e.g., influence of employment or nutrition, and have been rarely investigated [3, 25].

In this study, our aim was to provide information on hypoPT management, information needs, and the impact on daily living in patients with hypoPT in Germany.

## Methods

### Design

From 10/2020 to 10/2021, an online questionnaire, which was developed and pilot-tested with hypoPT patients, was available to patients with hypoPT. Information regarding the survey was distributed via the patient organization “Netzwerk Hypopara” or by their treating physician. The online survey was supported by the German Society of Endocrinology and the German Society of Nuclear Medicine. Upon request, a paper-based version of the questionnaire was available and provided to the patient. Detailed information regarding the design can be found elsewhere [26].

Eligibility criteria were a verified diagnosis of hypoPT by the treating physician and the condition needed to have been present for at least 6 months to be considered a chronic form. Due to the anonymity of the data collected, no ethics committee approval was necessary (confirmed by the Ethics Committee of the Landesärztekammer Rhineland-Palatinate).

### Assessments

All data were provided by the patients themselves through the online questionnaire. The average time to complete the questionnaire was 18 min. Time since diagnosis was documented using the following categories: 0.5–1 year, 1–2 years, 2–5 years, 5–10 years, and more than 10 years. Patients were asked for the cause of their hypoPT. If surgery was the cause, an additional question regarding the reason for surgery was asked.

For information on medication, patients provided the brand names of the medication they used for treatment of their hypoPT. Complementary medicine was assessed by a yes/no question followed by a free text question asking for details. Time intervals for monitoring were assessed using seven categories, as follows: *weekly, every 2–12 weeks, every 3–6 months, every 7–12 months, less than every 12 months, never,* and *don’t know.* Blood level parameters were provided by the patient if available and the patient was willing to share this information. The date of the blood level measurement was also obtained.

Patients were also asked whether they knew the treatment information card of the Netzwerk Hypopara (https://hypopara.de/cms/neuer-hypopara-patientinnen-und-patienten-pass/. [) and how useful they considered this passport to be. The treatment information card was developed in order to provide information for treating physicians in emergency situations. The information provided in the passport should help physicians who are not aware of the disease to make correct choices in critical situations such as severe hypocalcemia. The passport contains information regarding symptoms of hypo- and hypercalcemia, and patients can additionally add their last blood level parameters.

Patients stated whether information regarding hypo- and hypercalcemia was provided to them and, if information had been provided, the usefulness of this information was assessed using a four-point Likert-scale (“*not at all useful*” “*somewhat useful*”, “*quite useful*”, and “*very useful*”). For information needs, a five-point Likert-scale ranging from “*no information need*” to “*high information need*” was used. Patients were asked whether they had been hospitalized for hypocalcemia, hypercalcemia, a secondary disease, or complications. If a hospitalization had occurred, the total number of hospitalizations for the respective category (hypo-, hypercalcemia, secondary disease, or complications) was obtained.

The impact on daily living was divided into three groups, as follows: *psychological support, nutrition,* and *occupation.* For the assessment of *psychological support,* patients were asked if they required psychological support for coping with their disease and by whom (e.g., psychologist, psychotherapist) this support was provided. Nutritional impairments were obtained using a four-point Likert-scale (“*not at all”, “a little”, “quite a bit”,* and *“very much*”) and free text options where patients could state their impairments if they existed. The current employment status was assessed in four categories, as follows: *Employed/self-employed*, *retirement annuity (regular retirement), unable to work*, and *other.* The impact on occupation was assessed by asking if the disease had resulted in any changes to the working situation; if changes had occurred, *reduction of working hours, early retirement, being laid off/fired, change of occupation,* and *other* were the answering options. Working patients were asked about the number of days they had to miss work within the last 3 months due to their disease, and if they were going to work despite feeling unable to do so, and what their work capability (0–100%) was during these days.

To assess symptom burden, the HPQ-28 was used [27]. To categorize symptom burden, the mean value of all items (scores ranging from 0 to 3 per question) was calculated. Participants were categorized into low symptom burden (mean score 0 to < 0.5), medium symptom burden (mean score ≥ 0.5 to < 1.5), and high symptom burden (mean score ≥ 1.5).

### Statistical analysis

Descriptive statistics of the study population are given as mean values, median values, or percentages depending on the type of data. Univariate comparisons were performed using chi-square tests for categorical variables or Mann–Whitney-U tests for continuous data. All statistical analyses were performed using R (R version 4.0.4, R Foundation for statistical computing).

## Results

### Study population

Questionnaire data were provided by 268 patients. Four patients were excluded due to having had their hypoPT for less than 6 months and were therefore not considered chronic, possibly being instead transient. Thus, 264 patients with a mean age of 54.5 years (SD: 13.3) and 85.2% female were included in the analysis. The majority of patients (92.0%) reported that surgery was the cause of their hypoPT, and of those, 41.2% reported that surgery for thyroid cancer was the cause. More than half (54.9%) of the patients had received their hypoPT diagnosis more than 10 years ago. Details of the study population are presented in Table 1.

**Table 1:** Patient characteristics (n = 264) using mean (SD) or n (%)

Characteristic	
Age (mean (SD))	54.5 (13.3)
Sex (n (%))	
male	36 (13.6%)
female	225 (85.2)
missing	3 (1.1)
Education (n (%))	
below 10 years	36 (13.6)
10 years	102 (38.6)
more than 10 years	125 (47.3)
missing	1 (0.4)
Living situation (n (%))	
alone	61 (23.1)
with someone	203 (76.9)
Occupation (n (%))	
employed/self-employed	145 (54.9)
regular retirement	57 (21.6)
unable to work	45 (17.0)
other	17 (6.4)
Member of Self-help organization (n (%))	
no	167 (63.3)
yes	97 (36.7)
Time since diagnosis (n (%))	
6 months—1 year	6 (2.3)
1–2 years	22 (8.3)
2–5 years	47 (17.8)
5–10 years	43 (16.3)
more than 10 years	145 (54.9)
missing	1 (0.4)
Cause of HPT (n (%))	
non-surgical	21 (8.0)
* genetic/autoimmune*	10
* idiopathic*	6
* other*	5
surgical	243 (92.0)
* thyroid cancer*	100 (41.2)
* adenoma/goiter/nodules*	124 (51.0)
* hyperparathyroidism*	6 (2.5)
* hyperthyroidism*	10 (4.1)
* other*	3 (1.2)
Symptom burden (n (%))	
none to low	59 (22.3)
medium	75 (28.4)
high	130 (49.2)
Medication (n (%))	
calcium	153 (58.0)
calcitriol	152 (57.6)
alphacalcidol	23 (8.7)
colecalciferol	66 (25.0)
PTH	28 (10.6)
magnesium	49 (18.6)
dihydrotachysterol	31 (11.7)
missing	13 (4.9)

### Medication

Information on medication was provided by 251 patients (95.1%). Calcium (58.0%) and calcitriol (57.6%) were the most frequently used medications for hypoPT treatment among all patients. PTH replacement therapy was reported by 28 patients (10.6%), and 31 (11.7%) were using dihydrotachysterol for the treatment of hypoPT. Three patients reported the use of thiazides. Attempts to taper off medication were performed in 30.9% of all patients, with 33 (12.6%) having made more than one attempt. Complementary medicine for the treatment of their hypoPT was used by 13 patients (5.0%), with homeopathy and acupuncture being the most common methods.

### Monitoring und blood parameters

Almost 3/4 (73.8%) of all patients have their serum calcium levels checked at least every 6 months. Lower numbers were reported for phosphate (46.9%), magnesium (36.3%), creatinine (53.6%), and parathyroid hormone (49.6%) for the 6-month checks. For phosphate (17.2%), magnesium (14.5%), and creatinine (14.5%), a considerable number of patients did not know at what time interval their levels were checked. Calcium concentrations in 24-h urine were assayed in 36.0% of all patients on a yearly basis. No statistically significant differences regarding timing of the laboratory tests were seen when stratified by time since diagnosis. All information on monitoring intervals is presented in Table 2.

**Table 2:** Monitoring of blood levels by time since diagnosis

	Total				0.5–2 years since diagnosis				2–5 years since diagnosis				> 5 years since diagnosis				
	0–6 months	7–12 months	> 12 months	Don’t know	0–6 months	7–12 months	> 12 months	Don’t know	0–6 months	7–12 months	> 12 months	Don’t know	0–6 months	7–12 months	> 12 months	Don’t know	p-value
Calcium (blood serum)	194 (73.8)	57 (21.7)	10 (3.8)	2 (0.8)	25 (89.3)	2 (7.1)	1 (3.6)	0	39 (83.0)	5 (10.6)	2 (4.3)	1 (2.1)	129 (70.0)	50 (26.7)	7 (3.7)	1 (0.5)	0.1
Phosphate	123 (46.9)	42 (16.0)	52 (19.8)	45 (17.2)	17 (60.7)	4 (14.3)	4 (14.3)	3 (10.7)	26 (55.3)	2 (4.3)	9 (19.1)	10 (21.3)	79 (42.5)	36 (19.4)	39 (21.0)	32 (17.2)	0.1
Magnesium	95 (36.3)	29 (11.1)	100 (38.2)	38 (14.5)	15 (53.6)	3 (10.7)	8 (28.6)	2 (7.1)	22 (46.8)	3 (6.4)	15 (31.9)	7 (14.9)	57 (30.6)	23 (12.4)	77 (41.4)	29 (15.6)	0.2
Creatinine	140 (53.6)	42 (16.1)	45 (17.2)	34 (13.0)	17 (60.7)	5 (17.8)	3 (10.7)	3 (10.7)	26 (55.3)	3 (6.4)	9 (19.1)	9 (19.1)	96 (51.9)	34 (18.4)	33 (17.8)	22 (11.9)	0.4
Calcium (urine)	41 (15.7)	53 (20.3)	148 (56.7)	19 (7.3)	6 (21.4)	6 (21.4)	11 (39.3)	5 (17.9)	7 (14.9)	8 (17.0)	31 (66.0)	1 (2.1)	28 (15.1)	39 (21.1)	105 (56.8)	13 (7.0)	0.2
Parathyroid hormone	130 (49.6)	59 (22.5)	56 (21.4)	17 (6.5)	20 (71.4)	5 (17.8)	2 (7.1)	1 (3.6)	23 (48.9)	8 (17.0)	10 (21.3)	6 (12.8)	87 (46.8)	45 (24.2)	44 (23.7)	10 (5.4)	0.1

Data presented in n (%)

Half of the patients (n = 123) provided some information on blood test results. Blood samples were not taken on the same day the questionnaire was completed, so no association between current blood levels and results from the questionnaire can be investigated. Almost 2/3 (65.9%) of the patients with available data reported serum calcium concentrations above 2.15 mmol/l at their last measurement, while 12.1% reported levels below 2.0 mmol/l. All data on blood levels are presented in Table 3.

**Table 3:** Blood levels provided by 132 (50%) of the study participants

Calcium (blood) in mmol/l (n = 132)	n (%)
< 2.0	16 (12.1)
2.0 – 2.15	29 (22.0)
> 2.15	87 (65.9)
Phosphate in mmol/l (n = 102)	
< 0.84	6 (5.9)
0.84—1.45	58 (58.9)
> 1.45	38 (37.3)
Calcium-phosphate-product (n = 102)	
< 4.4	79 (77.5)
≥ 4.4	23 (22.5)
Vitamin D 25-OH in ng/ml (n = 79)	
< 30	25 (31.6)
30–70	33 (41.8)
> 70	21 (26.6)
Magnesium in mmol/l (n = 68)	
< 0.8	27 (39.7)
0.8 – 1.1	33 (48.5)
> 1.1	8 (11.8)
Creatinine in mg/dl (n = 110)	
≤ 0.9	58 (52.7)
> 0.9	52 (47.3)
Calcium (urine) in mg/d (n = 36)	
< 250 mg/d (f); 300 mg/d (m)	19 (52.8)
> 250 mg/d (f); 300 mg/d (m)	17 (47.2)

### Self-help and treatment information card

Being a member of a self-help organization was reported by 95 (37.1%) and 25.4% had attended a self-help group meeting to cope with their disease. The treatment information for patients with hypoPT from Netzwerk Hypopara [27] was known by 56.8% of the participants. The treatment information card was perceived as being “quite useful” or “very useful” by 93.3% of the patients. Whether their treating physician recommends the use of the treatment information card was answered by 38.9% with “yes” (34.2% “no” and 26.8% “don’t know”).

### Information

That their treatment was explained in detail to them was reported by 60.8% (160), while 39.2% of the patients (103) felt that it was not sufficiently explained. Participants reporting a detailed explanation stated that it was explained to them by their treating physician (61.4%), in the hospital (31.0%), by a patient organization (4.4%), or at other institutions (3.1%). Information on symptoms of hypocalcemia was provided for 71.8% (188) of the participants and 44.8% (117) for symptoms of hypercalcemia. For hypocalcemia, 84.1% of the participants who received information stated that this information was “quite useful” or “very useful”; similarly, 86.1% stated this for hypercalcemia symptoms. Data on information received stratified by time since diagnosis are shown in Table 4, with no statistically significant differences being observed between the groups.

**Table 4:** Information provided for patients with hypoparathyroidism

	Total	0.5-2 years	2-5 years	> 5 years	p-value
Treatment adequately explained					0.68
Yes		18 (64.3)	26 (55.3)	115 (61.5)	
No	103 (39.2)	10 (35.7)	21 (44.7)	72 (38.5)	
Information on hypocalcemia received					0.61
Yes					
No	74 (28.2)	7 (25.0)	11 (23.3)	56 (30.1)	
Information on hypercalcemia received					0.36
Yes					
no	144 (55.2)	12 (42.9)	26 (55.3)	106 (57.3)	

Data presented in n(%)

Univariate comparisons using chi-square tests

**Fig. 1:**
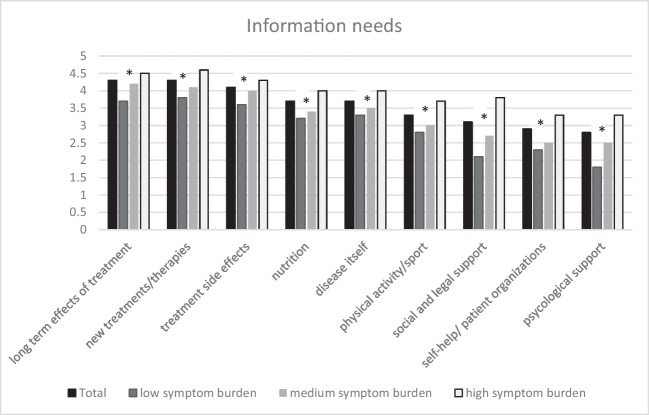
Mean scores of information needs stratified by symptom burden. * p < 0.005

Most unmet information needs were reported for *long-term effects of treatment* (mean: 4.3; 5.9% no information required), *new treatments/therapies* (mean: 4.3; 4.3% no information required), and *treatment side effects* (mean: 4.1; 7.8% no information required), followed by information on *nutrition* (mean: 3.7; 12.1% no information required), *the disease itself* (mean: 3.7; 8.3% no information required), *physical activity/sport* (mean: 3.3; 14.1% no information required), and *social and legal support* (mean: 3.1; 29.5% no information required). Information needs were least stated for *self-help/patient organizations* (mean: 2.9; 29.8% no information required) and *psychological support* (mean: 2.8; 32.3% no information required). In the subgroup of women below 40 years of age, a high information need regarding *pregnancy* (mean: 3.8; 15.0% no information required) was reported. Statistically significant differences in information needs stratified by symptom burden were observed for all information needs, with higher symptom burden being associated with higher information need (Fig. 1). No statistically significant differences regarding information needs stratified by reason for hypoPT (non-surgical vs. surgical (cancer) vs. surgical (other) were observed.

### Hospitalization

Participants reported hospitalizations due to hypocalcemia (31.6%), hypercalcemia (12.3%), and complications or secondary diseases (23.6%). For hospitalizations due to hypercalcemia, participants reported a median number of 1 (25%-quartile (Q0.25):1; 75%-quartile (Q0.75):2) hospital admission, while for hypocalcemia (Q0.25:1; Q0.75:2.75) and complications or secondary diseases (Q0.25:1; Q0.75:3) the median number of hospital admissions was two. The most pronounced complications or secondary diseases in the free text answers were kidney stones, heart problems, kidney problems, and mental health problems.

### Impact on daily living

To cope with their disease, 16.3% (43) of the participants made use of psychological services (e.g., psychologist, psychotherapist), with eight of those (18.6%) using more than one service. Impairments regarding regular food intake or nutrition were reported by 37.9% (102) of participants, with 17.9% reporting being “a little impaired”, 11.5% “quite impaired”, and 9.5% “very much impaired”. The main impairments were “phosphate intake,” “calcium intake,” “gastrointestinal problems,” and “generally not feeling well.” Being employed or self-employed at the time of the survey was reported by 145 participants (54.9%). Within the 3 months before the survey, 13.3% of the working participants stated that they had taken at least 1 day off from work due to hypoPT. Going to work despite feeling unable to within the last 3 months occurred in 52.7% of the participants currently working. The average work capability on these days was 47.3% (SD: 19; median 49%). Changes in the working situation due to hypoPT were stated by 79 participants (30.0%). The most frequent changes were “reduction of working hours” (41.8%), “early retirement” (26.6%), “being laid off/fired” (12.7%), “other” (12.7%), and “change of occupation” (6.3%).

## Discussion

Approximately 75% of the patients had their serum calcium concentrations measured at least every 6 months and approximately half of the participants reported measurement of phosphate, creatinine, magnesium, and parathyroid hormone on a regular basis. Patients with hypoPT in Germany report unmet information needs and impairments in daily living are experienced for among a moderate share of patients.

With a mean age of 54.5 years and 85.2% being female, our study population is line with other studies [3, 23, 28]. Regarding the cause of hypoPT, differences can be observed. With 92.0% stating surgery as the cause for their hypoPT, this number is higher than that reported in some studies [3, 23, 28], although in recent studies, high shares of surgical causes have also been reported [24, 29]. The high share of surgical causes and thyroid cancer being the reason for surgery might be due to the fact that patients were approached through a patient organization whose parent group is a patient organization for thyroid cancer.

Most of the patients in our study were receiving conventional treatment consisting of calcium supplements and/or calcitriol. This is in line with another study from Germany [30]. In the international context, differences in the conventional treatment are reported. The use of calcium and the doses of active vitamin D differ between the USA and European countries [3, 5, 6, 9, 14, 31]. Only three patients in our study reported the use of thiazides, even though it is recommended to reduce urinary calcium excretion in patients with hypercalciuria [8]. A higher number (14–30%) of patients taking thiazides were reported in other studies [22, 23, 30, 31]. We are not able to determine whether this number is really as low as this in our study population or whether the patients did not connect the use of thiazides to the treatment of hypoPT but rather to treatment for hypertension. Since August 1, 2021, dihydrotachysterol has not been available in Germany (https://www.endokrinologie.net/pressemitteilung/unterfunktion-nebenschilddruesen-standardmedikament-nicht-mehr-erhaeltlich.php [), which might be problematic since 11.7% of the patients in our study were treated with dihydrotachysterol and now need to change treatment. Almost 11% of the participants were treated with PTH. In the near future, these patients will have to go back to conventional treatment or hope for other new therapies since Takeda announced the end of NATPAR for 2024 [32]. Going back to conventional treatment will cause problems for these patients since they were treated with PTH and this is only done and such a change is typically only made if conventional treatment does not work.

Various guidelines and consensus statements recommend assessing blood serum levels of calcium, phosphate, magnesium, and creatinine at least every 6 months (3–12 months depending on the guideline) if the patient is on a stable treatment regimen [8, 10, 11, 33–35]; 24-h urine calcium excretion should be measured annually to identify hypercalciuria and other potential renal impairments [8, 10, 11, 33–35]. For calcium blood concentrations, an acceptable share of 73.8% of the patients in our study reported having measurements at least every 6 months. For phosphate, magnesium, and creatinine, approximately half of our patients reported monitoring frequencies consistent with the guidelines. Only 30.9% of the patients reported having at least yearly 24-h urine calcium excretion assessments, the lowest frequency of all measurements. In a study by Allemeyer et al. [13] in Germany, 76% of the hypoPT patients reported having regular calcium assessments. In a large US study by Hadker et al. [15], 76% reported having three or more calcium blood tests within the last 12 months. For Belgium and the Netherlands, 91% of physicians stated that biochemical parameters are monitored at least twice a year. In this study, 24 h urinary calcium excretion was monitored regularly by 59% of the physicians [16]. Astor et al. [3] stated that in Norway, 44–69% of the patients with hypoPT or pseudohypoparathyroidism never had their urine calcium measured. Regular monitoring is also important to minimize the risk of complications, symptom burden, and QoL impairments [17, 25, 27]. In our study, 36 patients provided information on calcium levels in urine, and approximately 50% of the patients who provided information here had elevated calcium urine concentrations. This might be seen as a hint that 24-h urine calcium excretion assessments should be monitored on a regular basis. Almost 50% of the participants stated that their PTH is checked on a regular basis. If patients are considered as having permanent hypoPT, it is quite surprising that PTH is measured on a regular basis. There may be several possible explanations. First, the possibility exists that parathyroid function recovers [36]; secondly, that the level of PTH values may help to estimate the remaining capacity to stimulate the enzyme 1-alpha hydroxylase to convert 25-OH-vitamit D3 to 1,25-vitamin D3; and thirdly, it is recommended in a guideline for children and young adults [33].

A treatment information card for patients with hypoPT might help them receive adequate treatment in emergency situations, such as severe hypocalcemia, and it may additionally provide physicians with information regarding the disease if they are not familiar with it. While a common European card for adrenal insufficiency is available [37] and many patients with this condition use the card [38, 39], emergency passports for hypoparathyroidism only exist on a national level in a few European states, such as Germany, Norway, and Sweden [14]. In Germany, the recommendation to use a treatment information card has found its way into guidelines for the treatment of thyroid diseases [40]; however, only 56.8% of the patients in our study knew about this passport. A possible explanation for this number might be the fact that physicians do not know about it. In line with findings from Astor et al. [14], an emergency passport is considered useful by hypoPT patients und might help in critical situations. Astor et al. [14] reported that the usefulness of an emergency passport was highest among hypoPT patients who had been hospitalized for hypo- or hypercalcemia. A total of 44% had been hospitalized for acute hypocalcemia, 16% for acute hypercalcemia, and 10% for hypo- and hypercalcemia. These numbers are slightly higher as compared to our study, with 31.6% being hospitalized for hypocalcemia, 1.32% for hypercalcemia, and 23.6% for complications or secondary disease. Rates of hospitalization vary across studies [16, 19, 22–24, 41], also depending on the population under study, with it not being uncommon that patients had more than one hospital admission during their disease course [15, 23].

Besides monitoring of patients, guidelines recommend that patients should receive information regarding treatment and should also be provided with information on hypo- and hypercalcemia [2]. A well-informed patient has the opportunity to deal better with hypo- and hypocalcemia and, therefore, a reduction of hospitalization or emergency department visits might be achieved [14]. In our study, 60.8% of the patients stated that their treatment was adequately explained to them. Hence, almost 40% do not have the feeling that they are well informed about their disease and treatment. This is in line with our result mentioned above, that the highest information needs were seen to be related to treatment and medication. According to Astor et al. [14], 21% of the patients stated that they did not receive adequate information about treatment and symptoms of their disease. Among our study population, information on hypo- and hypercalcemia was provided for 71.8 and 44.8%, respectively. These findings are in line with findings from Hadker et al. [15], where 56% of the patients agreed that “they felt unprepared to manage their condition.” These results show that patient and health care provider education is important [7, 11]. Special emphasis should be placed on physician education, since Cho et al. [42] have shown that the perception of the disease might differ between physicians and patients and that patients feel that physicians do not understand their disease [15]. This so-called empathy gap might negatively influence patients’ QoL [26]. Besides the treatment and disease-related information needs, patients in our study also reported high information needs for areas of daily living such as nutrition or sports. In our study, patients with a higher symptom burden reported higher information needs across all domains. Symptom burden has been found to be associated with impairments in QOL, although this is the first study investigating information needs and symptom burden. Our results again show that special attention should be given to patients with medium or high symptom burden [25, 26].

For nutrition, 37.9% of patients reported impairments in food intake or nutritional habits. To our knowledge, this is the first study assessing the subjective evaluation of impairments in food intake, while other studies have reported symptoms such as bowel problems or nausea [15, 27], which then might influence nutritional habits. Another important aspect which should be considered in the management of patients with hypoPT is the impact of the disease on the ability to work. In our study, 17.0% were unable to continue work and 30.0% mentioned changes in the work situation due to their disease. Astor et al. [3] reported for Norway that 40% of the hypoPT patients received permanent or temporary social security benefits. The ability to work could be an important issue for patients with hypoPT since it might influence QoL [26]. An aspect which also has an impact on daily living in patients with hypoPT is the influence of the disease on mental well-being. Various studies have reported a higher rate of depression and anxiety among patients with hypoPT [19, 30, 43–45]. Psychological support for coping with the disease was sought by 16.3% of the patients in our study. This rate is the same or even higher compared to cancer patients [46, 47].

### Strengths and limitations

This study has several limitations. All data were provided by the patients themselves and no validation through medical records was possible. Therefore, it is possible that patients do not know their blood concentration measurements or the exact number of hospitalizations, although it has been shown that patients with hypoPT do know about their received treatment and monitoring [15]. Nevertheless, it is important to be sensitive to the patients’ point of view regarding their management because it has been shown that the perception between the treating physician and the patient can differ significantly [15, 42]. Secondly, due to our use of a cross-sectional design, we were not able to observe longitudinal effects or changes during the treatment course. Moreover, no control group (e.g., patients after thyroid surgery without hypoPT) was available to examine whether changes exist. Büttner et al. [48] investigated the QoLin patients with and without hypoPT after treatment for thyroid cancer and found that the hypoPT group reported worseQoL, indicating that hypoPT patients might need closer monitoring. Additionally, due to the use of an online survey for data collection, there may be selection bias; however, since patients were also recruited through their treating physician, we believe that the sample was drawn from a representative population. We were not able to gather information on the specialty (e.g., endocrinologist, general practitioner) of the current treating physician. Nevertheless, guidelines for the treatment of hypoPT are not related to a specific medical specialty and in Germany patients with hypoPT are treated by a variety of experts. Ideally, patients would be treated by an endocrinologist; however, in Germany the majority are probably treated by their general practitioner or by a nuclear medicine specialist since these are often responsible for aftercare in thyroid cancer patients in Germany [49]. We are satisfied that the study population characteristics are in line with findings from other studies [3, 23, 28]. The strengths of our study are the large sample size despite the rarity of hypoPT and the heterogeneity of our study population. A broad set of questions, which were pilot-tested by patients, was used to obtain a good overview of the management, information needs, and impact on daily living of patients with hypoPT in Germany.

## Conclusion

This study is one of the first studies to assess the management, impact on daily living, and the information needs of patients with hypoPT in a large study sample.

Additionally, it has been shown that hypoPT might have an impact on activities of daily living. Patient and physician education regarding hypoPT is a key concept for improving the management of patients with hypPT.

## Data Availability

The datasets generated during and/or analyzed during the current study are not publicly available but are available from the corresponding author on reasonable request.
